# Significant expansion and red-shifting of fluorescent protein chromophore determined through computational design and genetic code expansion

**DOI:** 10.1007/s41048-018-0073-z

**Published:** 2018-11-10

**Authors:** Li Wang, Xian Chen, Xuzhen Guo, Jiasong Li, Qi Liu, Fuying Kang, Xudong Wang, Cheng Hu, Haiping Liu, Weimin Gong, Wei Zhuang, Xiaohong Liu, Jiangyun Wang

**Affiliations:** 1 Institute of Biophysics, Chinese Academy of Sciences, Beijing, 100101 China; 2 College of Life Sciences, University of Chinese Academy of Sciences, Beijing, 100049 China; 3 Key Laboratory of Physics and Technology for Advanced Batteries (Ministry of Education), Department of Physics, Jilin University, Changchun, 130012 China; 4 State Key Laboratory of Structural Chemistry, Fujian Institute of Research on the Structure of Matter, Chinese Academy of Sciences, Fuzhou, 350002 China

**Keywords:** Green fluorescent protein, Red-shift, Unnatural amino acids, Computational design

## Abstract

**Abstract:**

Fluorescent proteins (FPs) with emission wavelengths in the far-red and infrared regions of the spectrum provide powerful tools for deep-tissue and super-resolution imaging. The development of red-shifted FPs has evoked widespread interest and continuous engineering efforts. In this article, based on a computational design and genetic code expansion, we report a rational approach to significantly expand and red-shift the chromophore of green fluorescent protein (GFP). We applied computational calculations to predict the excitation and emission wavelengths of a FP chromophore harboring unnatural amino acids (UAA) and identify in silico an appropriate UAA, 2-amino-3-(6-hydroxynaphthalen-2-yl)propanoic acid (naphthol-Ala). Our methodology allowed us to formulate a GFP variant (cpsfGFP-66-Naphthol-Ala) with red-shifted absorbance and emission spectral maxima exceeding 60 and 130 nm, respectively, compared to those of GFP. The GFP chromophore is formed through autocatalytic post-translational modification to generate a planar 4-(*p*-hydroxybenzylidene)-5-imidazolinone chromophore. We solved the crystal structure of cpsfGFP-66-naphthol-Ala at 1.3 Å resolution and demonstrated the formation of a much larger conjugated π-system when the phenol group is replaced by naphthol. These results explain the significant red-shifting of the excitation and emission spectra of cpsfGFP-66-naphthol-Ala.

**Graphical abstract:**

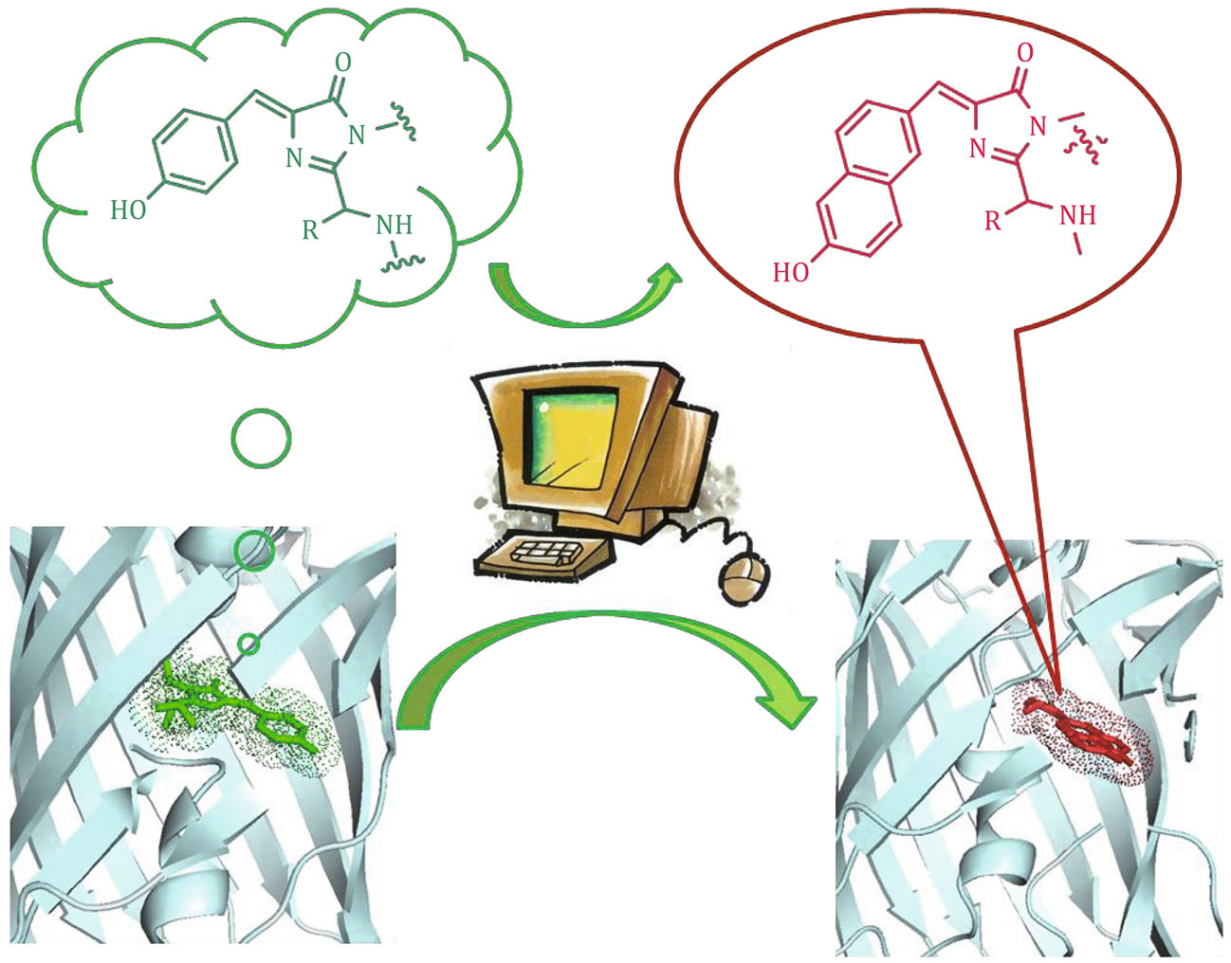

## Introduction

Fluorescent proteins (FPs) with far-red and infrared-region emission wavelengths are powerful tools for tracking protein localization in cells, cell migration, and deep-tissue imaging (Enterina *et al*. 2015; Nienhaus and Nienhaus 2014; Niu and Guo 2013; Sengupta *et al*. 2014; Shaner *et al*. 2011). Methods to achieve red-shifted FPs have evoked widespread interest and continuous engineering efforts (Chang *et al*. 2012; Enterina *et al*. 2015; Grimm *et al*. 2015; Newman *et al*. 2011; Niu and Guo 2013; Shu *et al*. 2009; Subach *et al*. 2011; Subach and Verkhusha 2012). Despite recent results, however, the design of these mutated FPs remains mostly dependent on high-throughput screening, which is a costly and tedious task. The development of modern computational chemistry allows a better understanding of the transition-state structure of FPs through theoretical calculation (Chica *et al*. 2010; Mou *et al*. 2015). Through this technology, key factors can be determined for the design of FPs with significantly red-shifted excitation and emission spectra. When the 66Tyr residue of the chromophore in various FPs is substituted with HqAla, excitation and emission spectra red-shifted by 30 nm are achieved (Liu *et al*. 2013). In the present work, a computation-based design and genetic code expansion are applied to rationally design an appropriate unnatural amino acid (UAA), 2-amino-3-(6-hydroxynaphthalen-2-yl)propanoic acid (naphthol-Ala), and genetically incorporate the same to the chromophore of cpsfGFP. cpsfGFP-66-naphthol-Ala has significantly red-shifted absorbance and emission spectral maxima compared to those of wild-type green FP (GFP). Structural analysis reveals the formation of a novel naphthol-imidazolinone (NapI) chromophore with a significantly larger conjugated π system compared to that of the original 4-(*p*-hydroxybenzylidene)-5-imidazolinone (HBI) chromophore in GFP. Furthermore, as a noteworthy and promising technology in super-resolution imaging, GFP and some of its mutants undergo efficient photoconversion into a red fluorescent state (Bogdanov *et al*. 2009; Elowitz *et al*. 1997; Saha *et al*. 2013). The oxidation red fluorescence of cpsfGFP-66-Naphthol-Ala revealed remarkably red-shifted excitation and emission spectra. This oxidation redding phenomenon was observed *in vivo* through confocal and fluorescence microscopy.

## Results and discussion

### Calculation and rational design of red-shift FP chromophore

**Fig. 1:**
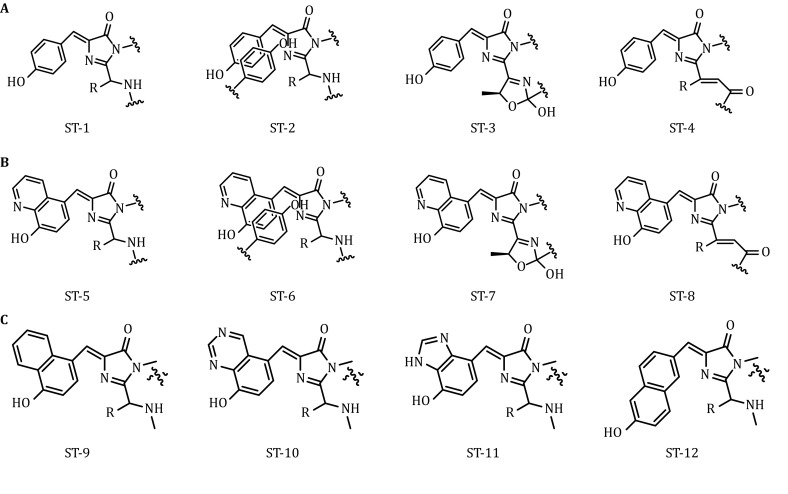
A ST-1, ST-2, ST-3, and ST-4, respectively, representing the chemical structures of sfGFP, sfYFP, PsmOrange, and eqFP650 containing the 4-(*p*-hydroxybenzylidene)-5-imidazolinone chromophore. **B** ST-5, ST-5, ST-7, and ST-8, respectively, representing the chemical structures of sfGFP-66-HqAla, sfYFP-66-HqAla, PsmOrange-72-HqAla, and eqFP650-67-HqAla containing the 8-hydroxyquinolin-imidazolinone chromophore. **C** Chemical structures of the predicted chromophores ST-9–ST-12

When the sfGFP 66Tyr site is substituted with a heterocyclic amino acid, the resulting excitation and emission spectra are red-shifted by approximately 30 nm (Liu *et al*. 2013). The fluorescence frequencies of the red-shifted chromophores (Fig. 1, ST-1–ST-8) were first computed by the time-dependent density functional theory (TDDFT) method with the long-range-corrected (LRC) exchange–correlation functional (Laurent and Jacquemin 2013). The gas-phase vertical absorption and emission energies of the eight chromophores (ST-1–ST-8 in Fig. 1), including their neutral and anionic states, are presented in Table 1. A trend of spectral shifting trend was well represented by the calculations. From left to right in each row of Table 1, the absorption and emission energies of either neutral or anionic chromophores are red-shifted compared to those of their left-hand neighbors. In addition, the absorption and emission energies of neutral and anionic chromophores in lower rows are red-shifted compared to the corresponding values indicated in upper rows. All frequency-change features observed were consistent with the experiment values.

**Table 1:** Calculated absorption and emission energies (eV) of chromophore models ST-1–ST-8 and their corresponding dipole moments (a.u.)

	ST-1		ST-2		ST-3		ST-4	
	*E* (∆*E*)	Dip.	*E* (∆*E*)	Dip.	*E* (*∆E*)	Dip.	*E* (*∆E*)	Dip.
Neutral								
*S*_0_ → *S*_1_	3.87	8.04	3.78 (− 0.09)	6.60	3.50 (− 0.37)	9.59	3.16 (− 0.71)	10.47
*S*_1_ → *S*_0_	3.27	9.28	3.16 (− 0.11)	8.01	2.93 (− 0.34)	11.49	2.69 (− 0.58)	12.98
Anionic								
*S*_0_ → *S*_1_	3.23	12.84	3.16 (− 0.07)	11.08	2.75 (− 0.48)	15.03	2.56 (− 0.67)	18.41
*S*_1_ → *S*_0_	3.04	12.76	2.95 (− 0.09)	10.67	2.59 (− 0.45)	15.41	2.43 (− 0.61)	18.80
Exp.			Abs. (− 0.15) Emi. (− 0.08)		Abs. (− 0.29) Emi. (− 0.24)		Abs. (− 0.46) Emi. (− 0.52)	

	ST-5		ST-6		ST-7		ST-8	
	*E* (∆*E*)	Dip.	*E* (∆*E*)	Dip.	*E* (*∆E*)	Dip.	*E* (*∆E*)	Dip.
Neutral								
*S*_0_ → *S*_1_	3.55 (− 0.32)	8.12	3.50 (− 0.37)	6.62	3.21 (− 0.66)	10.41	3.00 (− 0.87)	11.36
*S*_1_ → *S*_0_	3.03 (− 0.24)	9.75	2.94 (− 0.33)	8.08	2.72 (− 0.55)	12.80	2.49 (− 0.78)	14.42
Anionic								
*S*_0_ → *S*_1_	3.12 (− 0.11)	11.67	3.01 (− 0.22)	9.12	2.67 (− 0.56)	16.12	2.46 (− 0.77)	19.45
*S*_1_ → *S*_0_	2.94 (− 0.10)	11.19	2.83 (− 0.21)	9.17	2.52 (− 0.52)	16.75	2.34 (− 0.70)	20.07
Exp.	Abs. (− 0.25) Emi. (− 0.15)		Abs. (− 0.28) Emi. (− 0.19)		Abs. (− 0.42) Emi. (− 0.34)		Abs. (− 0.56) Emi. (− 0.61)	

“∆*E*” represents the energy red-shift of models ST-2–ST-8 compared to that of the ST-1 model. The experimental absorption and emission energy red-shifts of these models relative to those of the ST-1 model are also shown

The transition dipole moments of the chromophores also revealed a regular pattern. Table 1 shows that, in general, larger transition dipole moments corresponded to more red-shifted excitation and emission spectra. The transition dipole moment of emission was consistently larger than that of absorption. Intramolecular charge transfer significantly affects the spectra of many conjugated chromophores or polymers. Population analysis indicated that, besides large intramolecular charge transfers, a free electron flow channel can also decrease the transition (absorption and emission) energy.

The calculations provide a reliable guide for the design of red-shifted FPs. First, large conjugated π electrons afford a spacious and free electron flow place. Second, an ideal side chain should provide efficient electron-withdrawing and -donating effects. “Left-ring” type (HBI and HQI) charge transfers affect the anionic “right-ring to left-ring” charge transfer process more extensively than the neutral charge transfer process, and the neutral “left-ring to right-ring” charge transfer process is more influenced by the side chain of the “right-ring” charge transfer process than the “left-ring” transfer process. Third, in many conjugated polymer materials, N is a typical electron-withdrawing atom. In the case of HQI, elimination of N from HQI may promote the anionic “right-ring to left-ring” charge transfer process.

We predicted the structures of the heterocycle-containing chromophores ST-9–ST-12 (Fig. 1). Using the same calculation, the fluorescence frequencies of these chromophores were computed to design more red-shifted FPs. The gas-phase vertical absorption and emission energies of these chromophores (ST-9–ST-12 in Fig. 1) in their neutral and anionic states are presented in Table 1. Compared with the absorption and emission energies of the ST-1 chromophore in Table 2, those of the anionic ST-12 chromophore showed larger red-shifts.

**Table 2:** Calculated absorption and emission energies (eV) of the chromophore models ST-9–ST-12

	ST-9 *E* (∆*E*)	ST-10 *E* (∆*E*)	ST-11 *E* (∆*E*)	ST-12 *E* (∆*E*)
Neutral				
*S*_*0*_ → *S*_1_	3.51 (− 0.36)	3.56 (− 0.31)	3.72 (− 0.15)	3.60 (− 0.27)
*S*_1_ → *S*_0_	3.03 (− 0.24)	3.00 (− 0.27)	3.19 (− 0.08)	3.08 (− 0.19)
Anionic				
*S*_0_ → *S*_1_	3.09 (− 0.14)	3.06 (− 0.17)	3.21 (− 0.02)	2.72 (− 0.51)
*S*_1_ → *S*_0_	2.94 (− 0.10)	2.58 (− 0.46)	2.97 (− 0.07)	2.56 (− 0.48)
Exp.				Abs. (− 0.27) Emi. (− 0.49)

“∆*E*” refers to the energy red-shift of the four predicted chromophore models compared to that of the ST-1 chromophore model

### Synthesis and genetic incorporation of 2-amino-3-(6-hydroxynaphthalen-2-yl) propanoic acid (hereafter termed naphthol-Ala)

**Fig. 2:**
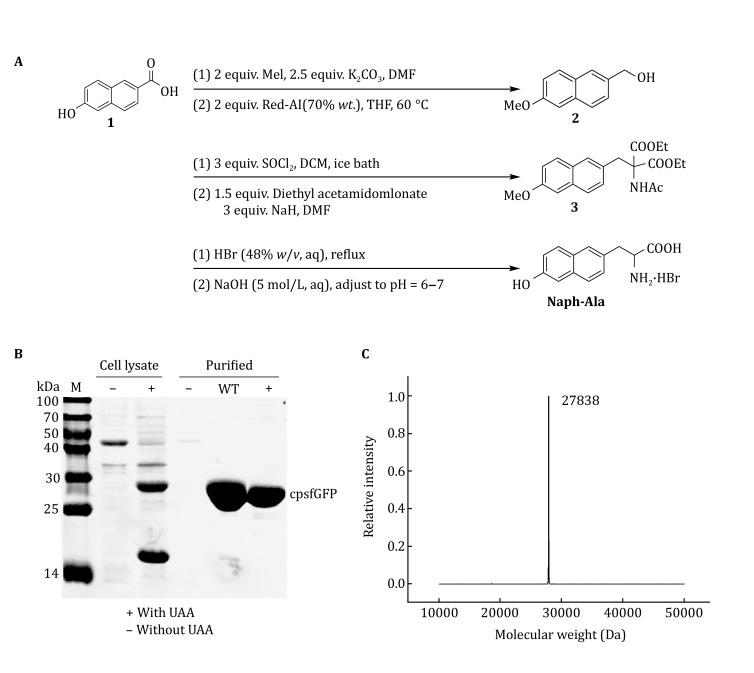
**A** Synthesis of 2-amino-3-(6-hydroxynaphthalen-2-yl) propanoic acid (naphthol-Ala). **B** Coomassie-stained SDS-PAGE gel indicating the expression of the cpsfGFP-66-naphthol-Ala mutant in the presence and absence of 1 mmol/L naphthol-Ala. **C** ESI–MS spectra of the TAG66 mutant of cpsfGFP, expected mass: 27873 Da, found: 27873.27 Da

We synthesized the UAA naphthol-Ala from the best candidate chromophore (ST-12) and genetically encoded it into cpsfGFP. Naphthol-Ala can be synthesized through six steps (Fig. 2A). In brief, 6-hydroxy-2-naphthoic acid is first methylated to yield methyl ester and then reduced to obtain a hydroxyl group through halogenation by sulfoxide chloride. The corresponding chloride is reacted with diethyl acetamidomalonate in alkaline conditions to form the ester precursor of the target UAA, which is then deprotected to form the hydrobromide salt of naphthol-Ala.

To deliver naphthol-Ala to the defined protein sites in *E. coli* through the TAG codon, a mutant *M. jannaschii* tyrosyl amber suppressor tRNA (*Mj*tRNA_CUA_^Tyr^)/tyrosyl-tRNA synthetase (*Mj*TyrRS) pair must be constructed. The desired *Mj*TyrRS clone was obtained after three rounds of positive selection and two rounds of negative selection from the *Mj*TyrRS library. This clone can resist 120 μg/mL chloramphenicol due to the expression of the chloramphenicol acetyl transferase (CAT) gene in the presence of 1 mmol/L naphthol-Ala but can only resist 20 μg/mL chloramphenicol in the absence of the UAA. The desired UAA RS was named naphthol-Ala RS. Sequencing of this clone revealed the following mutations (Table 3): Y32R, L65H, H70G, F108N, Q109C, D158N, and L162S. The Y32R and H70G mutations create additional space to accommodate the bulky naphthol group. L65, F108, and D158 are mutated to hydrophilic amino acids, thus creating a large binding pocket while providing additional hydrogen bonding interactions to stabilize the naphthol ring. Naphthol-Ala can also be incorporated into proteins with the following mutants (Chen and Tsao 2013): Y32E, L65T, D158S, I159A, H160P, Y161T, L162Q, A167W, and D286R in NpOH-RS1; and Y32E, L65V, K90E, I159A, H160W, Y161G, L162Q, A167I, and D286R in NpOH-RS2. The yield of the Z-domain mutant with NpOH-RS1 was roughly 7 mg/L culture in the presence of naphthol-Ala. These results demonstrate that the active site of tRNA (*Mj*tRNA_CUA_^Tyr^)/tyrosyl-tRNA synthetase has high capacity and variability for incorporating even the same UAA. To enhance the yield of the mutant proteins, naphthol-Ala RS was constructed on the pEVOL system.

**Table 3:** Sequence of naphthol-Ala-specific aaRSs

Position	32	65	70	108	109	158	162	164	200
Tyr RS	Y	L	H	F	Q	D	L	Y	D
Naphthol-Ala RS(8)	R	Y	G	N	C	N	S		

To determine the efficiency and fidelity of naphthol-Ala incorporation, an amber stop code was substituted for the Tyr66 site in cpsfGFP containing a C-terminal His6 tag. Protein expression was carried out in *E. coli* in the presence of the selected synthetase (naphthol-Ala RS), *Mj*tRNA_CUA_^Tyr^ with 0.2% arabinose, 1 mmol/L IPTG, and 1 mmol/L UAA. The protein expressed in the absence of the UAA was taken as the negative control. Analysis of the cell lysate and purified protein by Coomassie-stained SDS-PAGE showed that the full-length cpsfGFP mutant is expressed efficiently only in the presence of naphthol-Ala, thereby indicating the specific activity of naphthol-Ala RS for naphthol-Ala only (Fig. 2B). The yield of the Tyr66 mutant cpsfGFP (termed cpsfGFP-66-Naphthol-Ala) was 30 mg/L, whereas that of sfGFP was 100 mg/L. ESI–MS revealed that cpsfGFP-66-naphthol-Ala has an average mass of 27,873.27 Da (Fig. 2C), consistent with the calculated mass of 27,873 Da for the Y66 → naphthol-Ala cpsfGFP mutant, where the chromophore of the cpsfGFP mutant is fully matured after self-catalyzed dehydration and oxidation.

### Fluorescence characterization of cpsfGFP-66-naphthol-Ala

**Fig. 3:**
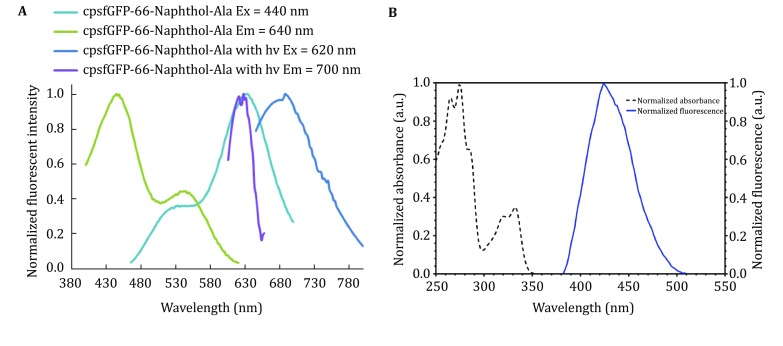
**A** Absorption and emission spectra of cpsfGFP-66-naphthol-Ala in 20 mmol/L MOPS-citrate buffer at pH 5 before and after 450-nm laser irradiation for 2 min in the presence of 1 mmol/L potassium ferricyanide. **B** Normalized absorption and emission spectra of 10 mmol/L naphthol-Ala in 60 mmol/L Tris–HCl buffer at pH 7, Ex,max = 331 nm; Em,max = 424 nm

We characterized the fluorescent property of cpsfGFP-66-naphtol-Ala, which shows red-shifted anionic state absorbance and emission spectral maxima at 545 and 640 nm, respectively (Fig. 3A, Table 4); these values are higher than those of sfGFP by 60 and 130 nm, respectively. The excitation and emission spectra of naphthol-Ala do not show any significant overlap with those of cpsfGFP-66-naphthol-Ala (Fig. 3B), thus indicating that the observed red-shift is not due to UAA small molecules.

**Table 4:** Summary of the key characteristics of fluorescent proteins

	cpsfGFP-66-naphthol-Ala		cpsfGFP-66-naphthol-Ala after 405-nm irradation	eqFP670
Excitation peak (nm)	450	545	620 nm	605
Emission peak (nm)	637	640	695 nm	670
Fluorescence QY	0.15	0.02	–	0.06
Molar extinction coefficient (l/(mol·cm)) at excitation maximum (pH 7.4)	82,600	12,700	–	70,000
Brightness^a^ (a.u.)	12,390	254	–	4200
Photostability, confocal^b^ (s)	–	7.5^c^	–	75
p*K*a	7.7	7.7	–	4.5
Reference	This work	This work	This work	Shcherbo *et al*. (2010)

^a^Calculated as the product of the molar extinction coefficient and quantum yield

^b^Time to bleach 50% of the brightness of the fluorescence signal

^c^Excitation wavelength is 561 nm

**Fig. 4:**
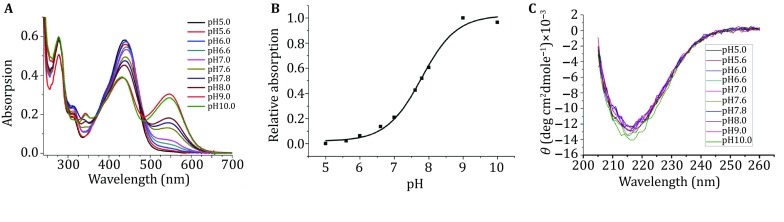
**A** UV–Vis spectra of cpsfGFP-66-naphthol-Ala in different pH value buffer. **B** Anionic chromophore fraction of cpsfGFP-66-naphthol-Ala at varying pH. **C** CD spectra of cpsfGFP-66-naphthol-Ala in the presence of buffers of various pH

We performed UV–Vis titrations of cpsfGFP-66-naphthol-Ala at various pH. Figure 4A shows that neutral cpsfGFP-66-naphthol-Ala displays a strong absorption peak at 450 nm and that new peaks corresponding to the anionic state appear at 545 nm with the increasing pH (p*K*a = 7.7). The CD spectra of cpsfGFP-66-naphthol-Ala from pH 5–10 show that the secondary β-sheet structure does not change with pH fluctuations, thereby indicating that the protein remains correctly folded throughout pH titration (Fig. 4B). These results are consistent with the results predicted by the computational calculations. The detected emission red-shift energy (− 0.49 eV) of cpsfGFP-66-naphthol-Ala is comparable with the calculated emission red-shift energy of the ST-12 chromophore, which has a ∆*E* value of − 0.47 eV (Table 2). The CD spectra of cpsfGFP-66-naphthol-Ala in the presence of buffers of various pH show no difference. (Fig 4C),

As potential tools in super-resolution imaging for investigating dynamic processes in living cells, GFP and some of its mutants undergo efficient photoconversion to a red fluorescent state. Under anaerobic conditions, the redding state of GFP shows excitation–emission maxima at 525 and 600 nm, respectively. In the presence of potassium ferricyanide, EGFP shows red-shifted fluorescence spectra with excitation and emission peaks at 575 and 607 nm, respectively (Bogdanov *et al*. 2009). We thus investigated whether cpsfGFP-66-naphthol-Ala can facilitate the oxidation redding process in the presence of potassium ferricyanide. After incorporating naphthol-Ala into the 66th position of cpsfGFP and irradiating the mutant protein using a 450-nm laser (100 mW/cm^2^) for 2 min in the presence of 1 mmol/L potassium ferricyanide, cpsfGFP-66-naphthol-Ala showed a remarkable red-shift with excitation and emission spectral maxima at 620 and 695 nm, respectively (Fig. 3A). These results indicate that oxidation redding can also occur in the presence of cpsfGFP-66-naphthol-Ala. Although the mechanism of GFP redding remains under investigation, it likely involves a two-electron oxidation process, where the amino acids near the chromophore, such as Glu222 (Saha *et al*. 2013), may act as an electron donor, and potassium ferricyanide acts as an electron acceptor. Upon electron transfer between these groups, a Ds-red-like chromophore is formed.

### Crystallography characterization of cpsfGFP-66-naphthol-Ala

**Fig. 5:**
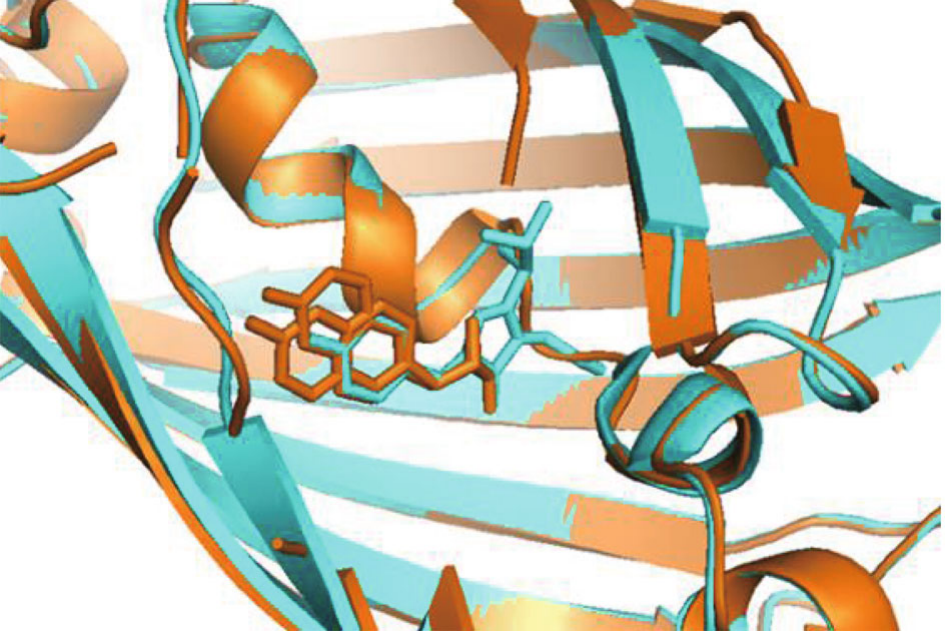
Alignment of sfGFP and cpsfGFP-66-naphthol-Ala. The chromophores of cpsfGFP-66-naphthol-Ala (*orange*) and sfGFP (*blue*) show that the naphthol or phenol and imidazolinone rings are clearly coplanar

To elucidate structural changes causing the red-shifted absorption and emission spectra of cpsfGFP-66-naphthol-Ala, we determined the structure of cpsfGFP-66-naphthol-Ala at 1.3 Å resolution. Figure 5 shows that substitution of Tyr66 by naphthol-Ala causes substantial crowding around the chromophore. The protein backbone of cpsfGFP-66-naphthol-Ala is highly similar to that of sfGFP. The 66th naphthol-Ala group presents two conformations due to the higher accessibility of the chromophore to solvents compared with sfGFP. The naphthol and imidazolinone rings are co-planar, thereby indicating that, similar to GFP, the chromophore is fully matured after nucleophilic attack of Gly67-Nα on the carbonyl group of Ser65, followed by dehydration and oxidation of the α–β bond in the naphthol-Ala group (Chudakov *et al*. 2010).

### Confocal and fluorescence microscopy of the oxidative redding state of cpsfGFP-66-naphthol-Ala

**Fig. 6:**
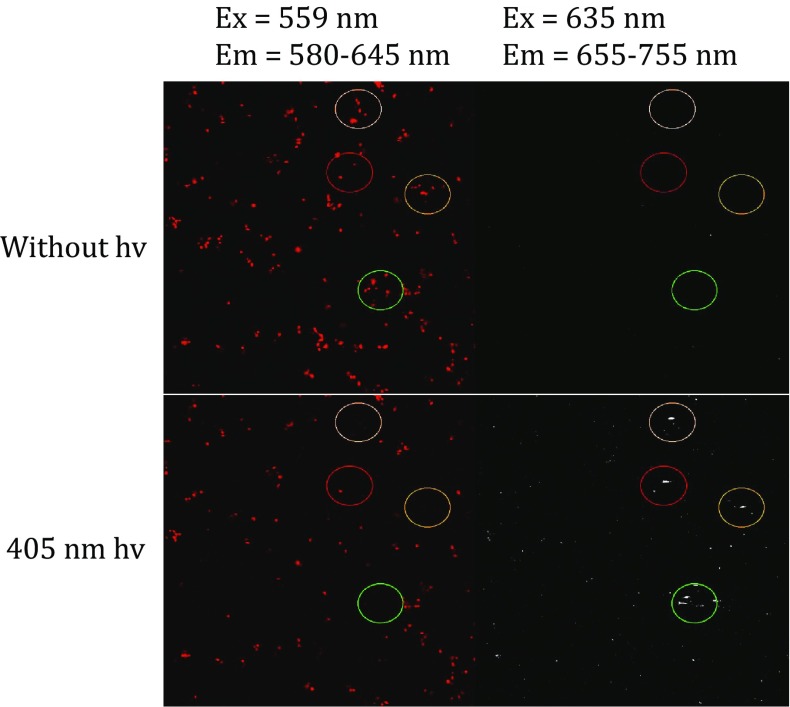
Pre- and post-photoconversion images of a thin (~10 μm) layer of *E. coli* cells expressing cpsfGFP-66-naphthol-Ala in 20 mmol/L MOPS-citrate buffer at pH 5 under 405-nm excitation delivered through a 1.2-NA objective to the region within the circle in bleaching mode (Red channel: 580–645 nm; Red channel: 655–755 nm). Photoconversion was performed at 37% laser power and 30 iterations for 111 ms

We tested whether the oxidative redding phenomenon of cpsfGFP-66-naphthol-Ala could be visualized through confocal and fluorescence microscopy *in vivo*. Before irradiation, a thin (~ 10 μm) layer of *E. coli* cells expressed with cpsfGFP-66-naphthol-Ala in 20 mmol/L MOPS-citrate buffer at pH 5 was prepared. The cells showed fluorescence at the detection window of 580–645 nm but were only slightly detectable at 655–755 nm. After irradiation with a 405-nm excitation laser for 2 min in the presence of 1 mmol/L potassium ferricyanide (highlighted by circles in Fig. 6), the fluorescence in the detection range of 655–755 nm increased. Overall, the oxidation redding phenomenon of cpsfGFP-66-naphthol-Ala could be observed *in vivo* through confocal and fluorescence microscopy.

## Conclusion

In conclusion, we demonstrated that a red-shifted FP could be designed via a rational computational design and genetic code expansion. Our work establishes a platform for further optimization of FPs with superior properties for deep-tissue and super-resolution imaging. The method presented in this work will benefit further studies on FP engineering.

## Experimental section

### Materials and reagents

6-Hydroxy-2-naphthoic acid was purchased from J&K chemical. All other chemicals used in this work were purchased from Sigma-Aldrich and applied without further purification. Silica gel chromatography purification was carried out using Silica Gel 60 (230–400 mesh). The PCR reagents, T4 DNA ligase, and restriction endonucleases were purchased from Fermentas. The Ni-resin (NTA) affinity purification reagents and column were purchased from Qiagen. Genes and primers were synthesized by Sangon Biotech.

### Instrument

All ^1^H-NMR spectra are reported in parts per million (ppm) and were measured relative to the signals of DMSO (2.5 ppm). ^13^C-NMR spectra are reported in ppm relative to those of residual DMSO (40 ppm). The mass spectra of the chemicals were obtained using a Waters LC–MS instrument equipped with a single-quadrupole mass detector and an electrospray ionization source (Waters ACQUITY QDa), while those of the proteins were obtained using Agilent 6100 equipment with a series of triple-quadrupole mass spectrometers (Agilent Technologies, CA, USA). Protein purification was performed using AKTA UPC 900 FPLC system (GE Healthcare), and absorption spectra were obtained at room temperature using a UV–visible spectrometer (Agilent 8453, Agilent Technologies). Fluorescence spectra were obtained using a microplate reader equipped with SkanIt 2.4.3 RE software for Varioskan Flash (Varioskan Flash, Thermo Fisher Scientific Inc.). Cell fluorescent images were recorded at room temperature by confocal and fluorescence microscopy (FV1000, OLYMPUS).

### Theoretical calculation

The chromophores used for the calculations were taken from a structural model (PDB: 4JFG) and built with manual modifications. The excited-state geometry of each chromophore was calculated using the TDDFT and LRC methods. The TDDFT method greatly reduces computational costs compared with MCSCF techniques, while the LRC method can deal with long-range electron correlations, which are common in π-conjugated fluorescent chromophore transitions, with greater ease than conventional hybrid functionals, such as B3LYP. In Filippi’s work, the LRC functionals CAM-B3LYP and LC-BLYP presented overall good agreement with the extrapolated results of solution experiments in vacuum conditions for wild-type GFP and wave function methods. All calculations were performed using the Gaussian 09 suite of programs (Frisch *et al*. 2016).

### Synthesis of (6-methoxynaphthalen-2-yl)methanol (2)

6-Hydroxy-2-naphthoic acid (1) (3.764 g, 20 mmol) was dissolved in anhydrous DMF (30 ml), after which K_2_CO_3_ (6.91 g, 50 mmol, 2.5 equiv.) and MeI (2.80 ml, 45 mmol, 2.25 equiv.) were added to their mixture. The reaction mixture was stirred vigorously for 48 h at room temperature, diluted with ice-water (300 ml), and extracted with ethyl acetate (3 × 100 ml). The organic phase was washed with brine and dried with Na_2_SO_4_, and the solvent was evaporated under reduced pressure to yield an off-white solid precipitate. This solid was dissolved in anhydrous THF (50 ml), after which a solution of sodium bis(2-methoxyethoxy) aluminum hydride (Red-Al, 70 *wt*%) in toluene (14.6 ml, 40 mmol, 2 equiv.) was added dropwise to it under a nitrogen atmosphere. The mixture was refluxed at 60 °C for 4 h and then quenched with water (2.5 ml). The supernatant was obtained by centrifugation, and the solvent was removed under reduced pressure. Flash column chromatography (petrol ether/ethyl acetate 1:1) formed compound 2 (3.61 g, 19.2 mmol, 96%) as a white solid.

### Synthesis of diethyl 2-acetamido-2-((6-methoxynaphthalen-2-yl)methyl) malonate (3)

Compound 2 (3.61 g, 19.2 mmol) was dissolved in anhydrous dichloromethane (30 ml) and then cooled in an ice-bath. Sulfoxide chloride (4.4 ml, 60 mmol, 3 equiv.) was added to the solution dropwise, and the mixture was stirred at 0 °C for 2 h. The solvent was removed, and the residue was co-evaporated twice with dichloromethane (20 ml) to yield a faint yellow solid.

A suspension of diethyl acetamidomalonate (6.26 g, 28.8 mmol, 1.5 equiv.) in anhydrous DMF (30 ml) was added to sodium hydride (1.38 g, 57.5 mmol, 3 equiv.) in small portions, and the mixture was stirred for 30 min under ice bath. Menaphthyl chloride dissolved in anhydrous DMF (30 ml) was then added dropwise to this solution. The mixture was stirred overnight at room temperature, diluted with ice-water (200 ml), and extracted with ethyl acetate (200 ml). The organic phase was washed with brine and dried with Na_2_SO_4_, and the solvent was removed under reduced pressure. The residue was purified by flash column chromatography (petrol ether/ethyl acetate 1:1) to yield compound 3 as a white solid (6.74 g, 17.4 mmol, 90.6%).

### Synthesis of 2-amino-3-(6-hydroxynaphthalen-2-yl) propanoic acid hydrobromide (naphthol-Ala·HBr)

Compound 3 (6.74 g, 17.4 mmol) was dissolved in hydrogen bromide (40 ml, 48% *w/v*, aq.) and then refluxed at 110 °C overnight under a nitrogen atmosphere. The red solution was cooled to room temperature and then neutralized with 5 mol/L NaOH. The red precipitate was filtered, and the residue was co-evaporated thrice with menthol (3 × 10 ml) to afford the hydrobromide salt of naphthol-Ala (5.16 g, 16.5 mmol, 94.5%).

MS(ESI): mass calculated for C_13_H_13_N_1_O_3_ requires *m/z*: 231.09, found [M + 1]^+^ *m/z* 232.19; [M + Na]^+^ *m/z* 254.07.

^1^H-NMR (500 MHz, D_2_O) δ: 7.60 (*d*, *J* = 9.0 Hz, 1H), 7.54 (*d*, *J* = 8.5 Hz, 1H), 7.50 (s, 1H), 7.15 (dd, *J* = 8.5, 1.8 Hz, 1H), 7.03 (*d*, *J* = 2.4 Hz, 1H), 6.96 (dd, *J* = 8.9, 2.5 Hz, 1H), 4.20 (dd, *J* = 7.6, 5.7 Hz, 1H), 3.14 (dd, *J* = 14.7, 5.7 Hz, 1H), 3.15–3.08 (m, 1H).

^13^C-NMR (126 MHz, D_2_O) *δ*: 170.35, 152.66, 132.87, 128.97, 127.85, 127.60, 126.80, 126.55, 117.51, 108.35, 53.37, 34.76.

### Plasmids and cell lines used

The plasmid pBK-lib-jw1 encodes a library of *Methanococcus jannaschii* tyrosyl tRNA synthetase (TyrRS) mutants randomized at residues Tyr32, Leu65, Phe108, Gln109, Asp158, and Leu162. Any one of Ile63, Ala67, His70, Try114, Ile159, and Val164 is also either mutated to Gly or kept unchanged. Plasmid pREP(2)/YC encodes *Mj*tRNA_CUA_^Tyr^, the CAT gene with a TAG codon at residue 112, the GFP gene under the control of the T7 promoter, and a Tetr marker. Plasmid pLWJ17B3 encodes *Mj*tRNA_CUA_^Tyr^ under the control of the *lpp* promoter and *rrnC* terminator, the barnase gene (with three amber codons at residues 2, 44, and 65) under the control of the *ara* promoter, and an Ampr marker. Plasmid pBAD/JYAMB-4TAG encodes the myoglobin gene of mutant sperm whale with an arabinose promoter and *rrnB* terminator, *Mj*tRNA_CUA_^Tyr^ with an *lpp* promoter and *rrnC*terminator, and a tetracycline resistance marker.

### Genetic selection of the mutant synthetase specific for naphthol-Ala

pBK-lib-jw1 consisting of 2 × 10^9^ TyrRS-independent clones was constructed using standard PCR methods. *E. coli* strain DH10B harboring the pREP(2)/YC plasmid was used as the host strain for positive selection. Cells were transformed with the pBK-lib5 library, recovered in SOC for 1 h, and washed twice with glycerol minimal media with leucine (GMML) before plating on GMML-agar plates supplemented with 50 μg/mL kanamycin, 60 μg/mL chloramphenicol, 15 μg/mL tetracycline, and 1 mmol/L naphthol-Ala. The plates were incubated at 37 °C for 60 h, the surviving cells were scraped, and the plasmid DNA was extracted and purified by gel electrophoresis. pBK-lib-jw1 DNA was then transformed into electro-competent cells harboring the negative selection plasmid pLWJ17B3, recovered for 1 h in SOC, and then plated on LB-agar plates containing 0.2% arabinose, 50 μg/mL ampicillin, and 50 μg/mL kanamycin. The plates were incubated at 37 °C for 8–12 h, followed by a similar extraction of pBK-lib5 DNA from the surviving clones. The library was then carried through a subsequent round of positive selection, followed by negative selection and a final round of positive selection (with 70 μg/mL chloramphenicol). At this stage, 96 individual clones were selected, suspended in 50 ml of GMML in a 96-well plate, and replica-spotted on two sets of GMML plates. A set of GMML-agar plates was supplemented with 15 μg/mL tetracycline; 50 μg/mL kanamycin; 60, 80, 100 or 120 μg/mL chloramphenicol; and 1 mmol/L naphthol-Ala. Another set of identical plates without naphthol-Ala was produced with 0, 20, 40, or 60 μg/mL chloramphenicol. After 60 h of incubation at 37 °C, one clone was found to survive at 100 μg/mL chloramphenicol in the presence of 0.5 mmol/L naphthol-Ala, and at 20 μg/mL chloramphenicol in the absence naphthol-Ala.

### Generation of mutants and site-directed mutagenesis analysis

Plasmid pET22b containing cpsfGFP was used to generate mutant cpsfGFPY66amber. Mutagenesis was confirmed through DNA sequencing analysis.

### Expression and purification of cpsfGFP-66-naphthol-Ala

To express mutant cpsfGFP, plasmid pET22b-cpsfGFP-66-TAG was co-transformed with pEOVL-naphthol-Ala synthetase (RS) into BL21(DE3) *E. coli* cells. Cells were amplified in 5 ml of LB media supplemented with 50 µg/mL ampicillin and 30 µg/mL chloramphenicol. A starter culture (1 ml) was used to inoculate 100 ml of liquid LB supplemented with appropriate antibiotics and 0.5 mmol/L naphthol-Ala. The cells were then grown at 37 °C to an *OD*_600_ of 1.1, and protein production was induced by the addition of 0.2% arabinose and 1 mmol/L IPTG. After 12 h, the cells were harvested by centrifugation and then sonicated. The supernatant was collected and incubated with Ni–NTA agarose beads for 1 h at 4 °C, filtered, and washed with wash buffer containing 50 mmol/L HEPES (pH 7.5), 500 mmol/L NaCl, and 20 mmol/L imidazole. The protein was eluted with the wash buffer containing 50 mmol/L HEPES, pH 7.5, 500 mmol/L NaCl, and 250 mmol/L imidazole. cpsfGFP-66-naphthol-Ala was loaded onto a MonoQ column and eluted with a NaCl gradient. cpsfGFP-66-naphthol-Ala was further purified by a Superdex 75 column (GE Healthcare) in buffer containing 20 mmol/L HEPES–NaOH (pH 7.5) and concentrated to 16 mg/mL. Protein concentration was quantified using the Bradford assay.

### Structural determination of cpsfGFP-66-Naphthol-Ala

Crystallization of cpsfGFP-66-naphthol-Ala was achieved through vapor diffusion against 100 mmol/L Tris (pH 6.0) and 20% PEG3K at 4 °C. The crystals appeared within 3 d and were flash-frozen in liquid nitrogen. Diffraction data were collected at a wavelength of 0.979 Å at BL17U of the Shanghai Synchrotron Radiation Facility with a Q315CCD detector. Data processing and reduction were carried out by using the HKL2000 package. The initial structure of cpsfGFP-66-naphthol-Ala was determined by molecular replacement with Molrep from the CCP4 suite by using the atomic coordinates of wild-type sfGFP (PDB code: 2B3P) as the search model. Molecular replacement solutions were modified and refined with alternative cycles of manual refitting. Structural refinement was carried out using PHENIX. During refinement, the Coot tool in the CCP4 program suite was used for the model building, ligand and water location, and real-space refinement of side chains and zones. The final structure of cpsfGFP-66-naphthol-Ala was checked for geometrical correctness with PROCHECK. The collected data and structural refinement statistics are summarized in Table 5. Cartoons and other protein structure representations were generated using PyMOL (http://www.pymol.org), and the atomic coordinates and structure factors were deposited in Protein Data Bank.

**Table 5:** Summary of data-collection and refinement statistics

Space group	*P*2_1_
Unit-cell parameters (Å)	*a* = 46.8, *b* = 49.3, *c *= 49.2
Resolution range (Å)	39.1–1.25 (1.29–1.25)
Number of unique reflections	58,574
Data completeness (%)	99.7 (100)
〈*I*〉/〈σ(*I*)〉	17.7 (2.8)
*R*_merge_ (%)^a^	6.4 (42.5)
*R*-factor/*R*_free_ (%)^b^	19.4/21.9
r.m.s.d. bond length (Å)	0.007
r.m.s.d. bond angles (°)	1.276
Number of atoms modeled	2102
Number of water molecules	215
Mean *B* factor (Å^2^)	16.2
Protein main-chain atoms	14.4
Protein side-chain atoms	16.1
Water molecules	25.3
Ramachandran plot statistics	
Residues in most favored region (%)	96.0
Residues in additional allowed region (%)	3.6
Residues in disallowed region (%)	0.4

^a^$R_{\text{merge}} \, = \,\sum_{hkl} \sum_{i} {{\left| {I\left( {hkl} \right) - \left\langle {I\left( {hkl} \right)} \right\rangle } \right|} \mathord{\left/ {\vphantom {{\left| {I\left( {hkl} \right) - \left\langle {I\left( {hkl} \right)} \right\rangle } \right|} {\sum_{hkl} \sum_{i} {\text{I}}\left( {hkl} \right),{\text{ where}}\,\,\left\langle {I\left( {hkl} \right)} \right\rangle }}} \right. \kern-0pt} {\sum_{hkl} \sum_{i} {\text{I}}\left( {hkl} \right),{\text{ where}}\,\,\left\langle {I\left( {hkl} \right)} \right\rangle }}$ is the main value of *I*(*hkl*)

^b^$R{\text{{-}factor}}\,{ = }\,\,{{\sum {\left| {\left| {F_{obs} } \right| - \left| {F_{calc} } \right|} \right|} } \mathord{\left/ {\vphantom {{\sum {\left| {\left| {F_{obs} } \right| - \left| {F_{calc} } \right|} \right|} } {\sum {\left| {F_{obs} } \right|} }}} \right. \kern-0pt} {\sum {\left| {F_{obs} } \right|} }},$ where *F*_obs_ and *F*_calc_ are the observed and calculated structure factors, respectively

The free *R* factor was calculated using 5% of the reflections omitted from the refinement. Numbers in parentheses represent the value of the highest resolution shell

### UV spectra of cpsfGFP-66-Naphthol-Ala

7 μmol/L solution of purified mutant cpsfGFP-66-naphthol-Ala protein was added to different NaiP/citric acid buffers of various pH, and their UV–Vis spectra were recorded using a quartz cuvette (100 μl, 1 cm path) at room temperature with Agilent 8453 UV–visible spectrophotometer.

### Excitation and emission spectra of cpsfGFP-66-naphthol-Ala

The fluorescence spectra of a 2 μmol/L solution of purified mutant cpsfGFP-66-Naphthol-Ala protein were obtained using a Thermo Varioskan Flash instrument equipped with SkanIt 2.4.3 RE software.

### Circular dichroism (CD) experiments

10 μmol/L solution of purified mutant cpsfGFP-66-naphthol-Ala in 10 mmol/L NaiP/citric acid buffer at various pH was transferred into a quartz cuvette (200 μl, 1 cm path), and spectra were obtained at room temperature by using a CD spectrometer (Chirascan Plus). Scanning was done thrice at a scan speed of 120 nm/min and 1-nm intervals. Data smoothing was not performed.

### Confocal and fluorescence microscopy

Pre- and post-photoconversion images were obtained from a thin (~10 μm) layer of *E. coli* cells expressing the target protein in MOPS-citrate buffer (pH 5) using 405-nm excitation delivered using a 1.2-NA objective to the region within the circle in bleaching mode (Red channel: 580–645 nm; Red channel: 655–755 nm; scale bar: 50 μm). Photoconversion was performed at 37% laser power and 30 iterations at 111 ms. All observations were obtained by confocal and fluorescence microscopy (FV1000, Olympus).

For photostability determination, samples were obtained by overnight induction of cpsfGFP-66-naphthol-Ala and eqFP650 expressed in the BL21 strain. Bacterial solution (1 ml) was centrifuged for 4 min at 4000 r/min, and the pellet obtained was washed thrice with PBS buffer and suspended in 1 ml of PBS buffer. Thereafter, 100 μl of this solution was placed on a glass that had been preprocessed with polylysine. After 30 min of incubation, the glass was washed with 300 μl of PBS buffer for microscopic observation. The samples were photobleached by 30 W/cm^2^ of 561 nm light, and the time was recorded. All observations were obtained using an Olympus 71 microscopy.
